# Flavivirus Receptors: Diversity, Identity, and Cell Entry

**DOI:** 10.3389/fimmu.2018.02180

**Published:** 2018-09-26

**Authors:** Mathilde Laureti, Divya Narayanan, Julio Rodriguez-Andres, John K. Fazakerley, Lukasz Kedzierski

**Affiliations:** ^1^Department of Microbiology and Immunology, Peter Doherty Institute for Infection and Immunity, University of Melbourne, Melbourne, VIC, Australia; ^2^Faculty of Veterinary and Agricultural Sciences, University of Melbourne, Melbourne, VIC, Australia

**Keywords:** flaviviruses, Japanese encephalitis virus, Zika virus (ZIKV), dengue virus, yellow fever virus, entry receptor

## Abstract

Flaviviruses are emerging and re-emerging arthropod-borne pathogens responsible for significant mortality and morbidity worldwide. The genus comprises more than seventy small, positive-sense, single-stranded RNA viruses, which are responsible for a spectrum of human and animal diseases ranging in symptoms from mild, influenza-like infection to fatal encephalitis and haemorrhagic fever. Despite genomic and structural similarities across the genus, infections by different flaviviruses result in disparate clinical presentations. This review focusses on two haemorrhagic flaviviruses, dengue virus and yellow fever virus, and two neurotropic flaviviruses, Japanese encephalitis virus and Zika virus. We review current knowledge on host-pathogen interactions, virus entry strategies and tropism.

## Introduction

The *Flavivirus* genus consists of more than 70 small, positive-sense, single-stranded RNA viruses transmitted by arthropods, in particular mosquitos and ticks. These include globally important human pathogens such as West Nile virus (WNV), Japanese encephalitis virus (JEV), dengue virus (DENV), Murray Valley encephalitis virus (MVE), tick-borne encephalitis virus (TBEV), Yellow Fever virus (YFV), and Zika virus (ZIKV). These viruses are responsible for some of the most severe arbovirus infections affecting humans, pose a serious threat to global health and have the potential to cause severe outbreaks. These are exemplified by the global distribution of DENV (1), the recent ZIKV outbreak in South America (2), YFV outbreaks in Africa (3), and Brazil (4) and the spread of WNV across North America (5). Flavivirus infections range from asymptomatic, through mild fever and arthralgia to life threatening haemorrhagic or encephalitic diseases (6). Flaviviruses are also able to persist in patients and can be responsible for long-term morbidities (7). There are no antiviral treatments for flavivirus infection currently in clinical use, and despite licensed vaccines against several of the viruses including YFV, JEV, TBEV, or DENV, outbreaks still occur highlighting challenges in implementing effective vaccination programs (8).

The flaviviral genome of ~11 kb contains a single open reading frame flanked by untranslated regions, and encodes 3 structural proteins (C, M, and E) and 7 non-structural proteins (NS). The mature virion features a surface densely covered with E glycoproteins and M proteins and a core consisting of capsid (C) protein and the RNA genome (9, 10). The entry into the target cell is dependent on E protein contact with its cognate receptor. E protein initially binds to attachment factors such as glycosaminoglycans. This effectively increases viral density on the cell surface, leading to high affinity receptor binding (11). The E protein ectodomain consists of three domains (E-DI, E-DII, E-DIII) of which E-DIII is thought to interact with attachment factors and receptors (12). E-DIII domain's importance is highlighted by the fact that a vast majority of potent, neutralizing antibodies has been mapped to this region. Nevertheless, anti-DI and DII antibodies, although less potent, show broader cross-reactivity and form a major pool of anti-E protein specific immonoglobulins (13). Receptor binding is followed by clathrin-mediated endocytosis (14), which is considered to be a major mechanism of flavivirus cell entry, although there are exceptions described below. This leads to formation of endosomes and low pH dependent changes in the E glycoprotein with subsequent membrane fusion and release of nucleocapsid into the cytosol (15). *In vitro*, flaviviruses are able to infect a plethora of cell lines originating from rodents, non-human primates, humans and mosquitos. However *in vivo*, fewer cell types seem able to support flavivirus replication (16). A wide range of cell surface receptors has been implicated in flavivirus entry into different cells types (11). Amongst the entry receptors postulated to be involved in flavivirus entry, the best characterized to date include α_v_β_3_ integrins (17, 18), C-type lectin receptors (CLR) (19–23), phosphatidylserine receptors TIM (T-cell immunoglobulin and mucin domain) and TYRO3, AXL and MER (TAM) (24). Recent studies indicate that flaviviruses can produce a range of structurally different virions. This structural heterogeneity may expand tissue tropism and ability to infect different cell types both in invertebrate and vertebrate hosts (25).

Flaviviruses are deposited into the skin epidermis by a mosquito bite where they encounter cells permissive to infection such as keratinocytes and skin dendritic cells (Langerhans cells) (26). Dendritic cells in particular appear to be a common initial target for flaviviruses. When infected, dendritic cells migrate to lymphoid organs where viral replication takes place allowing for flavivirus dissemination into circulation and internal organs (12). Viruses such DENV (27), JEV (28), ZIKV (29) have been shown to infect skin dendritic cells, and although there are no reports on YFV infecting Langerhans cells, it can nevertheless infect other types of dendritic cells (30). This interaction is mediated by DC-SIGN for JEV (28) and ZIKV (29), but appears to be DC-SIGN independent in case of DENV (31) and YFV (30).

Many flaviviruses are neuroinvasive and neurovirulent and cause central nervous system (CNS) damage (32). Neuroinvasive infections are observed with JEV, TBEV, and WNV (33, 34), and occasionally with haemorrhagic viruses including DENV (35). There is a paucity of knowledge regarding factors involved in CNS cell entry. While CLRs and TIMs are expressed by cells of the CNS (36–38), they are not expressed by neurons (39–41). However, members of the TAM family of receptors are expressed by different neuronal subtypes (42), though they are dispensable for ZIKV infection as ZIKV was able to infect and replicate in TAM receptor knockout mice (43).

As natural vectors, mosquitos and ticks are highly permissive to flavivirus infection. The virus can replicate in a range of arthropod tissues and cells (44, 45). Given that flaviviruses have only one glycoprotein, it seems likely that the mechanism of entry into vertebrate and invertebrate cells is evolutionarily conserved. A number of the cellular receptors implicated in flavivirus entry into mosquito cell lines overlaps with those identified for mammalian cells (46). Some flaviviruses are more selective regarding their arthropod host than others. For example, DENV is spread mainly by *Aedes spp*. mosquitos (6), WNV by *Culex spp*. (47), whereas JEV is transmitted by *Aedes, Anopheles* and *Culex spp*. (48). There appears to be a more restricted receptor repertoire used by flaviviruses for insect cell entry compared to mammalian cell entry. The range of clinical manifestations of flaviviral infection in the mammalian host suggests that these viruses may use a wide range of receptors. Mammalian tissues in general offer much greater range of receptors compared to invertebrates.

Identification of flavivirus entry receptors, particularly those involved in CNS infection, could lead to identification of novel therapeutic targets. For this review we will focus on four major flaviviruses of humans—DENV, JEV, ZIKV, and YFV, and discuss the differences and similarities in their mechanisms of entry into arthropod and mammalian cells.

## Dengue virus

DENV is one of the most common mosquito-borne viruses, mainly transmitted by *Aedes aegypti* mosquitoes, and occasionally by *Ae. albopictus*. Symptoms of DENV infection range from fever and muscle and joint pain (Dengue fever) to potentially life threatening haemorrhagic fever or shock syndrome. While DENV was endemic in <10 countries in the 1970's, it is presently a threat in over 128 countries and is responsible for almost 400 million human infections every year. About 24% of infections manifest in severe clinical symptoms. There is currently no treatment for DENV serotypes. There are four virus serotypes, and recovery from one serotype provides lifelong homologous immunity (49).

DENV is an icosahedral particle of 50 nm with a positive, single-stranded RNA genome of 10–11 kb (50). As in other flaviviruses, E protein is involved in receptor binding and fusion (51) and has the ability to bind to a wide range of cellular receptors to initiate DENV entry. The E-DIII domain has a role in cellular recognition (52) and has been suggested as a target for the development of a DENV vaccine (53).

Over the years, several cell membrane receptors involved in DENV entry have been identified. These include carbohydrate molecules (54–56), lectins (57, 58), and claudin-1 cell receptors (59). Carbohydrate molecules such as glycosaminoglycans (GAGs), sulphated polysaccharides, and glycosphingolipids (GSL) are widely expressed cell surface co-receptors for DENV entry and are believed to enhance viral entry efficiency. The highly sulphated form of GAGs, the heparan sulfates (HS) and heparan sulfates proteoglycans (HSPG), are essential for cellular adhesion to extracellular matrix and binding of polypeptide growth factors involved in intracellular signaling (56). It has been suggested that DENV first contacts HSPG, and that this weak interaction facilitates binding of virus to other receptors, which then results in virus internalization (55). Several studies have shown that pre-treatment with heparin can reduce or block DENV-2 infection (60, 61). However, the efficiency of inhibition of viral entry was dependent on numerous factors, such as the virus strains and the target cell (61). GSLs, a member of the same family of carbohydrate molecules as HS, are ubiquitous cellular components of eukaryotic plasma membranes that can also facilitate entry and binding of virus (54). However, GSLs are not required for DENV entry as the virus was able to enter GSL-deficient cells (62).

Cellular C-type lectin receptors (CLRs) are part of the host immune response to fungal, bacterial and viral infections (57). CLRs in mammalian cells include DC-SIGN/L-SIGN, mannose receptors (MR), and CLEC5A. DC-SIGN receptors are widely known because of their association with HIV. These receptors are also involved in DENV binding and internalization into dendritic cells (63). MR has been found to be the primary DENV cell receptor in macrophages. The CLEC5A receptor cooperates with DC-SIGN or MR to increase DENV binding and stability (58).

Other studies have suggested claudin-1 as a putative cell receptor for DENV entry through a direct interaction with the viral prM protein. Claudins are vital components of tight junction complexes and are essential for normal permeability of the epithelia (59, 64). DENV-2 entry was significantly reduced in claudin-1 deficient cells (59). Also, it has been demonstrated that caudin-1 is upregulated early in infection in order to facilitate entry and downregulated in late stage of infection (64).

Protein binding assays and mass spectrometry analysis have identified several additional potential flavivirus cellular receptors (65–67). Among them, the tubulin and tubulin-like proteins in C6/36 *Ae. albopictus* cell line (65). Heat shock proteins (HSPs) of ~70 kDa and 80 kDa were also identified as cellular receptors for all four DENV serotypes in C6/36 cell line (66, 68). HSPs are chaperone proteins involved in the regulation of folding and unfolding of cellular, and upon infection, viral proteins (69). The 70 kDa protein, also known as heat shock cognate protein (HSC70) or HSPA8, acts as a chaperone protein during DENV entry (70, 71). Modulation of HSC70 expression was observed during DENV-2 infection, with an increase on the cell membrane during infection, suggesting that DENV-2 utilizes HSC70 for entry into mosquito cells (67). In addition to its role in viral entry, HSP70 is involved in virion biogenesis and RNA replication (71). It appears that all four DENV serotypes are dependent on this chaperone protein family, which makes HSP70 proteins an interesting target for the design of a tetravalent DENV therapy or vaccine (71).

HSP90, another heat shock protein, can also act as chaperone. This protein interacts with six DENV proteins (69). While the involvement of HSP70 and HSP90 in DENV binding to host cells has been reported (70–72), these proteins are not involved in internalization of virus into the host cell (73).

As mentioned before, TIM/TAM family receptors have been implicated in flavivirus entry. DENV express on its surface phosphatidylserine (PS) and phosphatidylethanolamine (PE) molecules. Both PS and PE are known to directly interact with TIM/TAM receptors and DENV is able to enhance its entry by exploiting these interactions (74).

After binding to cellular receptors, internalization of viral particles occurs. For DENV, internalization occurs via pH-dependent endocytosis. Several endocytosis pathways are currently known, but clathrin-mediated endocytosis is the main pathway for DENV (75). The DENV use of clathrin-mediated endocytosis was demonstrated in C6/36 mosquito cells by biochemical inhibition of cell receptors (76), and in several human cell lines through siRNA silencing of genes associated with clathrin-mediated receptors (75, 77, 78). While this inhibition and specific gene silencing resulted in a decrease in viral load, a complete inhibition was not achieved, suggesting the existence of alternative entry pathways in mosquito and mammalian cells.

**Figure 1:**
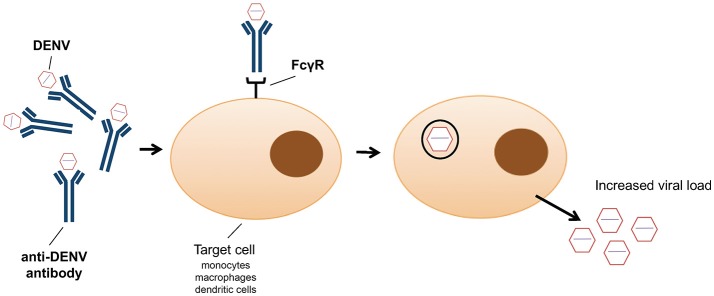
Schematic representation of antibody dependent enhancement (ADE) entry into monocytes, macrophages and dendritic cells as employed by dengue virus.

In addition to the exploitation of clathrin-mediated endocytosis, the host immune system can also promote viral entry (79). This phenomenon, known as antibody-dependent enhancement (ADE), was first described in 1964 by Hawkes (80) for WNV and JEV, and observed for DENV more than a decade later (81). Antibody-virus complexes are internalized by phagocytosis via Fc gamma receptors (FcγR) into macrophages, monocytes and dendritic cells (82) (Figure 1), thus facilitating virus entry (83). It has recently been shown that ADE increases membrane fusion activity, promoting DENV entry (79). Moreover, prM antibodies have the capacity to convert non-infectious, immature DENV particles into infectious particles and enhance their infectivity to levels comparable to wild-type virus (50). ADE has been linked to the observation that one flavivirus infection can enhance another (84). However, a recent study showed that ADE is dependent on the level of neutralizing antibodies, particularly IgG and IgM (85); only patients with a low level of neutralizing antibodies showed enhancement of DENV infection (85). Antibodies, even at low concentration, against the EDIII domain were able to block viral entry of the four DENV serotypes without inducing antibody-dependent enhancement (86). However, high IgG titres were observed in patients with ADE following DENV infection, in particular IgG1 levels were the highest in patients with dengue fever or shock syndrome (87). ADE has been recently identified as consequence of sensitisation with Dengvaxia quadrivalent vaccine, leading to severe vaccine-enhanced disease resulting in hospitalization (88).

## Japanese encephalitis virus

Globally, Japanese encephalitis is the most clinically important arboviral encephalitis, with an estimated annual prevalence of up to 50,000 cases (89). As is the case with most arboviral encephalitic infections, humans are dead-end hosts unable to develop a sufficiently high viremia to transmit to feeding mosquitos. The majority of JEV infections are asymptomatic. Approximately a third of clinical cases are fatal and half of survivors have neurological or neuropsychiatric sequelae with symptoms resembling Parkinsonian movement disorders, poliomyelitis-like paralysis or impaired cognition (90). Disease is most common in children up to 14 years of age. JEV has been expanding its endemic areas in Asia (91) and poses an unpredictable and emerging global threat.

In humans, JEV has been found in different anatomical compartments and a variety of cell types is able to support its replication. These include endothelial cells, granulocytes, dendritic cells, macrophages and cells of the CNS including astrocytes, neurons and microglia (92). The virus spreads from dermal tissues (93) to lymphoid organs (94) and during the acute stage of infection can be found in blood (95). Although highly neuroinvasive, the mechanism of JEV entry into the CNS is unclear. Transport along the olfactory nerve and across the blood brain barrier have been implicated in JEV invasion of the CNS (96, 97). Studies in rodent models indicate that the blood brain barrier is disrupted following neuroinvasion (98), and might be a consequence of invasion rather than an entry route. Once in the brain, JEV can infect pericytes (99), astrocytes (100) and microglia (101), and has a predilection for developing neurons and neuronal progenitors (102, 103). As described below, a number of receptors mediate entry into different cells types. The distinctive neuronal tropism suggests the existence of JEV-specific receptors in the CNS, but their nature remains elusive (104).

*In vitro* studies on mouse neuroblastoma cells indicate heat shock protein (HSP) 70 as a putative entry receptor present on neuronal cells (105). This has not been corroborated by *in vivo* experiments, but in human hepatoma Huh7 cells, HSP70 is required for entry (106). Recently, a member of the HSP70 family, glucose-regulated protein (GRP) 78, has been implicated in JEV entry into Neuro2a and BHK-21 cells (107, 108). In addition to HSP70 and GRP78, HSP90β also interacts with E protein and may be used by JEV to enter mammalian cells (109). Another member of the HSP70 family is heat shock cognate (HSC) protein 70. HSC70 has been suggested to be a receptor for entry into mosquito cells (110). HSC70 isoform D is essential for clathrin-dependent endocytosis of JEV into C6/36 cells (111). Clathrin dependence seems to be critical for JEV entry into mammalian cells with the exception of neuronal cells (112), where JEV internalization into rodent neuroblastoma cell lines has been shown to be clathrin-independent (113, 114) and independent of HSP70 family proteins. JEV can enter human neuronal cells by caveolin-mediated endocytosis (115), a process that is receptor-independent (116). Interestingly, JEV has been shown to utilize the dopaminergic signal transduction pathway to increase neuronal susceptibility to infection (117). Infection of human dopaminergic neuroblastoma cells *in vitro* leads to increased levels of secreted dopamine and activation of the phospholipase C cascade. The latter enhances formation of structures known as focal adhesions on the cell surface and increases JEV binding and entry. One of the main components of focal adhesions is αvβ3 integrin that recruits vimentin to the cell surface (118), and is involved in JEV binding and infection of BHK-21 cells (18). Vimentin is a putative JEV receptor (119, 120). Thus, by signaling through dopamine D2 receptors and activating the phospholipase C cascade, JEV induces recruitment of surface molecules that enhance and propagate infection in adjacent cells. Enhanced infection of dopaminergic neurons also explains why JEV is predominantly found in brain areas rich in these cells including the thalamus and the midbrain (121, 122). Whereas JEV infection of neurons may be most directly relevant for disease, other cells types are also likely to have an important role in the disease process. Microglial cells may be a viral reservoir due to long-term, high level of virus production in these cells (123). CD4 has been identified as a major receptor for JEV entry into microglia (124). Presumably, CD4 can be used by JEV to enter other CD4 positive cells such as T cells, macrophages or dendritic cells. Published data are scarce, however, JEV productive infection of splenic macrophages and T cells has been reported in a mouse model of infection (125). T lymphocytes have also been reported as a reservoir of latent JEV in asymptomatic children following recovery from acute infection (126). The involvement of CD4 in microglial cell entry has not been reported for any other flavivirus, however CD4 is the main receptor for retroviral entry and is primarily localized in lipid rafts (127). As mentioned above, HSP70 in lipid rafts is involved in JEV entry into human cells and in general lipid rafts play a critical role in JEV entry (128, 129). Moreover, lipid rafts, as well as clathrin-coated pits and caveolae, contain sphingolipids such as sphingomyelin (SM) that is involved in JEV attachment and entry (130). Studies in SM synthase 1 deficient mice infected with JEV showed a reduction in disease, indicating a role for SM in JEV infection models (130).

Despite advances in identification of new receptors associated with JEV entry and its clear tropism for neuronal cells in the CNS, the identity of a specific neuronal receptor remains elusive. Notably, JEV has the ability to infect cells in the absence of above mentioned putative receptors although at a reduced rate (104). This suggests that the entry process involves multi protein interactions with high degree of redundancy and a single, specific entry receptor might not exist. Alternatively, the inability to identify such a receptor highlights the limitations of *in vitro* systems commonly used to investigate virus-cell interactions.

## Zika virus

ZIKV is a mosquito-borne emerging pathogen that poses significant public health concerns due to recent rapidly expanding outbreaks. ZIKV was relatively unknown until 2007, when an outbreak occurred in Yap Island (Micronesia) (131). The virus was first isolated in the Zika Forest in Uganda from a rhesus monkey in 1947. In 1948 a second isolate from *Ae. africanus* mosquitoes was obtained from the same forest (132). Prior to the recent serious outbreak in French Polynesia, New Caledonia, the Cook Islands and Easter Island in 2013 and 2014 (133), Zika has not been reported to cause significant disease. Data from French Polynesia during the ZIKV epidemic documented the occurrence of Guillain-Barre syndrome and other neurological complications (134). The pathogenesis of ZIKV infection is poorly understood and involves a multifaceted interaction between viral and host factors. ZIKV has shown a significant tropism to the CNS and causes neurodegeneration, particularly of neural progenitor cells (135–137). ZIKV is also the only flavivirus known to have teratogenic effects in humans, including microcephaly, intracranial calcification and fetal death (138). As a result, the World Health Organization announced in 2016 that the ZIKV outbreak was a health emergency of international concern (139). Like other flaviviruses, ZIKV likely enters host cells through endocytosis instigated by an interaction of E glycoprotein with cell surface receptors. Identification of the entry receptor(s) for ZIKV is essential to understanding viral tropism and pathogenesis, and could lead to the development of novel therapeutics to treat the infection.

The first barrier for the virus to enter the host cell is the skin epidermis. ZIKV is transmitted by *Aedes* spp. mosquitoes, which deposit virus in the epidermis and dermis during the blood meal. Both dermal fibroblast and epidermal keratinocytes are permissive to ZIKV infection as are skin dendritic cells. Several entry receptors including the innate immune receptor DC-SIGN, transmembrane protein TIM-1 and TAM receptors (TYRO3, AXL, MER), have been shown to facilitate entry and enhance ZIKV infection (29). RNA silencing of TIM-1 and AXL in subsets of human skin cells showed a significant reduction in ZIKV titre in AXL knockdown, and in double AXL and TIM-1 knockdown, indicating that AXL is a major receptor for ZIKV entry at least in human skin cells. However, a recent study (43) investigating different infection routes of ZIKV, including subcutaneous, transplacental, vaginal, and intracranial infections in wild-type and TAM receptor null mice, showed no difference in viral titres. TAM receptors, at least in mice, are therefore not essential for ZIKV infection. Interestingly, WNV infection of neurons can be enhanced in mice lacking AXL and MER. This increase in infectivity was associated with changes in blood brain barrier permeability (140), suggesting that AXL and MER do not serve exclusively as receptors and might have other roles in WNV infection of the brain.

To reach the fetal brain, ZIKV must first be transported to the fetal circulation, and cross the placental barrier. The placental barrier is composed of placental barrier cells, trophoblasts and fetal endothelial cells, which separate the fetal blood in capillaries from maternal blood. ZIKV has been reported in the amniotic fluid of fetuses in Brazil (141). This observation strengthened the association of ZIKV with microcephaly in neonates. Moreover, it has been shown that microcephaly caused by maternal viral infection in mice could result from direct viral infection of the fetus via the trans-placental route as well as from a placental inflammatory response that affects fetal development (142). ZIKV can efficiently infect fetal endothelial cells, whereas WNV and DENV do not, highlighting ZIKV unique tropism among flaviviruses (143). These differences between flaviviruses are due to ZIKV ability to efficiently use AXL receptor to enter fetal endothelial cells (143).

TIM-1 was also observed to have an important role in placental entry of ZIKV (144). ZIKV was able to infect different human primary placental cell types and explants from chorionic villi. AXL, TYRO3, and TIM-1 were present in the primary placental cells and are found at the uterine-placental interface. Particularly high expression of TIM-1 has been observed in cells where maternal blood perfused placenta including basal decidua and neighboring chorionic villi. Expression of AXL and TYRO3 varied with explant donor, gestational age and cell type. Specific pharmacological inhibition of TIM-1 by duramycin (145) could inhibit ZIKV infection at the uterine-placental interface, indicating that TIM-1 is a putative receptor for ZIKV placental cell entry. However, the role of AXL, variation in the expression of AXL and TYRO3 in pregnant women and whether TIM-1 is the sole receptor for ZIKV infection of the placenta need further study.

Numerous studies on ZIKV infection in mice having defective interferon signaling, including IFNα/β knockout mice (146, 147), double knockout of IFNα/β and IFNγ (148), and triple knockout of IRF-3,-5,-7 (136, 149) showed viremia, microcephaly and death in young mice and viremia with recovery in adult mice. However, cell death and reduced proliferation was observed for adult neural stem cells (136) suggesting possible long term effects in adult brain followin ZIKV infection. ZIKV also infects other cell types, especially in the eye. ZIKV-inoculated mice develop ocular defects including conjunctivitis, pan uveitis, and infection of the optic nerve, cornea, iris, and ganglion and bipolar cells in the retina (150). AXL is expressed at high levels in retinal progenitor cells (151) suggesting a possible role in ZIKV infection of ocular cells. However, the ocular abnormalities were shown to be independent of AXL or MER, given that AXL^−/−^, MER^−/−^, and AXL^−/−^ MER^−/−^ double knockout mice sustained levels of infection similar to those of control animals. Nevertheless, AXL might have a role in ZIKV infection of glial cells via Gas6 mediated activation of AXL kinase (152).

*In vitro* and *in vivo* systems to study ZIKV infection of neural cells have been developed. ZIKV neuro-infection models using cultured neural precursor cells (NPCs), cortical organoids, mouse brains, and human fetal brain materials have been studied (153). Microcephaly in these models is associated with inflammation, reduced proliferation of NPCs and neuronal cell death. ZIKV-BR can infect mouse fetuses and infection of pregnant mice also causes disease in embryos with intrauterine growth restriction, including signs of microcephaly (154). This study also demonstrated that ZIKV-BR infects human cortical progenitor cells, increasing the rate of cell death. ZIKV was found to directly infect human NPCs with high efficiency providing a plausible explanation for the observed developmental phenotypes and associated teratogenicity in the neonatal brains (137). Based on previous studies (29, 151, 152), AXL is a strong candidate receptor for the entry of ZIKV into cells of the developing brain. The potential role of AXL to facilitate ZIKV infection of the neonatal brain was explored by determining, at a single cell level, RNA expression profiles in the developing human cerebral cortex (151). The study revealed a higher expression of AXL in the radial cells and neural stem cells of the developing brain throughout neurogenesis and in capillaries and astrocytes. However, loss of AXL expression following CRISPR/Cas9 gene editing, did not affect ZIKV infectivity into hNPCs or cerebral organoids (155).

As is the case with JEV, the identity of the receptors involved in ZIKV receptor mediated endocytosis remains to be elucidated. There might be tissue specificity in receptor mediated viral entry, with variation in receptor repertoire in the skin, placenta, neurons, and other cell types. Alternatively, given ZIKV unique ability to cross the placenta and infect developing neurons in the fetal brain, there might be some as yet unidentified receptors facilitating this process.

## Yellow fever virus

YFV is the prototype and namesake virus of the *Flavivirus* genus; *flavi* means yellow in Latin. When infecting humans YFV replicates in liver, heart, kidneys, and lungs causing a broad spectrum of clinical symptoms. These vary from asymptomatic infection to renal and hepatic failures with severe haemorrhagic disease (156). A live attenuated YFV vaccine 17D was created over 70 years ago and has been used safely in over 500 million people. The parent strain of 17D is the virulent Asibi strain (157) isolated in Africa in 1927. 17D was passaged more than 230 times in mouse and chicken embryonic tissue. The adaptation of 17D to grow in tissue culture resulted in loss of viscerotropism, neurotropism, and mosquito tropism (156), making it an ideal candidate for a vaccine. The genome of both strains has been sequenced (158). The extensive passage history gave rise to 68 nucleotide mutations and 32 amino acid substitutions. Most of the genetic differences occur in the envelope (E) protein gene (157). Interestingly, the molecular determinants and mechanisms of this attenuation remain largely unknown. It has been suggested that the differences in the E protein and its involvement in cell entry are determinants of the difference in pathogenicity between the 17D and Asibi strains (159, 160). Mutations in the E gene have been suggested to allow the 17D strain to bind and enter hosts cells more efficiently.

YFV shares genome organization and entry by clarithin-mediated endocytosis (CME) with most of the other flaviviruses. During YFV infection, the E protein binds to an unknown entry receptor that traffics the virion to endosomes. Similarly to other flaviviruses, increase in acidification of the endosome results in conformational changes in the E protein, membrane fusion and nucleocapsid release into the cytoplasm (156). The vaccine strain uses a clathrin- and caveolin-independent, but dynamin-2-dependent, pathway for infection (160). Dynamin-2 is a GTPase involved in cleaving off endocytic vesicles from the plasma membrane (161). The entry pathway of the 17D strain was further characterized as Rac1, Pak1, and cortactin independent (160). Clarithin-independent entry has been reported to mediate the internalization of a variety of viruses, such as rotavirus, human rhinovirus, influenza, and interestingly, JEV vaccine strain in neuronal cells (114, 162–164). Cells infected with 17D have been found to produce more viral RNA and INF-β, IL-29, ISG56, CCL5, and CXCL10 mRNA than those infected with the parental Asibi strain. In addition, 17D infected cells secrete INF-β, whereas cells infected with the Asibi strain do not. Virus entry through a clathrin-independent pathway allows for more efficient virion delivery into endosomes or protection from degradation, relative to entry via the classical clathrin-mediated route. This former entry route has been suggested to allow for a higher amount of viral RNA released into the cytoplasm (160). Viral RNA in the cytoplasm is detected by RIG-I, MDA5 and TLR7 (165), triggering strong innate immune responses. The Asibi strain on other hand, replicates at lower levels and inhibits the innate immune system. This difference in entry mechanism has been suggested to account for the differences in cytokine response between the two YFV strains, though further mutations in other proteins, such as NS2A could also be involved.

## Conclusions

Glycoprotein E is responsible for receptor-mediated attachment of flaviviruses to the host cell and membrane fusion. Although E protein of different flaviviruses share approximately 40% sequence identity (e.g., DENV and TBEV), their overall structural features are almost identical and this is assumed to apply to all flaviviruses (166). Cell entry is facilitated by a conserved peptide of 16 amino acids, located in E-DII region of the envelope glycoprotein (167). This conservation, coupled with highly organized conformational changes upon exposure to low pH (168), suggests evolutionary constraints allowing flaviviruses to enter both mammalian and arthropod cells. Yet, flavivirus receptors show diversity and significant cell type specificity. It is not unusual that a single molecule can bind to variety of targets as exemplified by immunoglobulins. However, their diversity and specificity are governed by V(D)J recombination, while the flaviviral glycoprotein E is conserved. The flavivirus infection is a consequence of multiple complex interactions between the virus and the target cell. It is clear that the flavivirus can exploit different endocytic routes that can be either clathrin or caveolae dependent or independent. The neurotropism of specific flaviviruses raises the question, is there a single specific neuronal receptor? What is the identity of this receptor and is the same receptor being used by all encephalitic flaviviruses? Another unresolved question is whether all flaviviruses share the same features of infection in the developing brain, or whether viruses such as microcephaly-causing ZIKV, exhibit a different infection pattern. It is also relevant to note that the expression of entry receptors (e.g., CLRs or TAM) does not account for flavivirus tropism and cellular models lacking those receptors are still permissive to infection. Identifying the relevant entry receptors is essential to deciphering the mechanisms of pathogenesis, tropism and viral biology. A better understanding of those processes will uncover new strategies for designing therapeutics and vaccines against flaviviruses.

## Author contributions

ML, DN, JR-A, and LK wrote sections of the manuscript, JF and LK edited and critically evaluated the manuscript.

### Conflict of interest statement

The authors declare that the research was conducted in the absence of any commercial or financial relationships that could be construed as a potential conflict of interest.
